# Comparative Transcriptome Analysis and Genetic Methods Revealed the Biocontrol Mechanism of *Paenibacillus* *polymyxa* NSY50 against Tomato *Fusarium* Wilt

**DOI:** 10.3390/ijms231810907

**Published:** 2022-09-18

**Authors:** Nanshan Du, Hui Guo, Ruike Fu, Xiaoxing Dong, Dongqi Xue, Fengzhi Piao

**Affiliations:** 1 College of Horticulture, Henan Agricultural University, Zhengzhou 450002, China; 2 Henan Key Laboratory of Fruit and Cucurbit Biology, Henan Agricultural University, Zhengzhou 450002, China

**Keywords:** tomato *Fusarium* wilt, *P. polymyxa* NSY50, *SlNAP1*, plant-growth-promoting rhizobacteria

## Abstract

*Fusarium* wilt caused by *Fusarium oxysporum* f. sp. *lycopersici* (*Fol*) is a common disease that affects tomatoes, which can cause the whole plant to wilt and seriously reduce the production of tomatoes in greenhouses. In this study, the morphological indexes, photosynthetic performance and incidence rate of NSY50 under *Fol* infection were evaluated. It was found that NSY50 could improve the growth of tomato seedlings and significantly reduce the incidence rate of *Fusarium* wilt. However, the molecular mechanism of NSY50 that induces resistance to *Fusarium* wilt is still unclear. We used transcriptomic methods to analyze NSY50-induced resistance to *Fol* in tomatoes. The results showed that plant defense related genes, such as *PR* and *PAL*, were highly expressed in tomato seedlings pretreated with NSY50. At the same time, photosynthetic efficiency, sucrose metabolism, alkaloid biosynthesis and terpene biosynthesis were significantly improved, which played a positive role in reducing the damage caused by *Fol* infection and enhancing the disease tolerance of seedlings. Through transgenic validation, we identified an important tomato NAC transcription factor, *SlNAP1*, which was preliminarily confirmed to be effective in relieving the detrimental symptoms induced by *Fol*. Our findings reveal that *P. polymyxa* NSY50 is an effective plant-growth-promoting rhizosphere bacterium and also a biocontrol agent of soil-borne diseases, which can significantly improve the resistance of tomato to *Fusarium* wilt.

## 1. Introduction

Tomato (*Solanum lycopersicum*) is an important vegetable crop that is widely cultivated in China and around the world. The growth process of tomatoes is easily disturbed by a variety of biotic and abiotic stresses, resulting in a significant decrease in their annual yield and quality [1]. In the process of greenhouse tomato cultivation, due to the high multiple cropping index, high temperature and humidity and relatively closed environment, it is easy to reduce the soil biodiversity and increase the pathogen quantity, eventually leading to serious leaf, fruit and soil-borne diseases [2]. Among them, *Fusarium* wilt is one of the most widely spread soil-borne fungi caused by *Fusarium oxysporum*, an ascomycete fungus belonging to the Nectriaceae family [3,4]. The genus *Fusarium* is widespread across the world, including more than 20 species, of which 14 species, such as *F. avenaceum*, F. equiseti, F. oxysporum, F. semitectum and F. solani, can infect vegetables and fruits, including potatoes, peppers, eggplants, melons, onions, pineapples, beans and bananas. Most *Fusarium* species are common in tropical and subtropical regions, and some in temperate regions. It is worth noting that *F. avenaceum*, *F. culmorum*, *F. graminearum*, *F. poae*, *F. cerealis*, *F. sambucinum* and *F. tricinctum* can infect cereals (barley, malt, oats, rye, corn, rice and wheat), beans, nuts and sugar beets, resulting in canker, root rot, brown foot rots, pink ear rot and wilts. It can be said that *Fusarium* is a serious threat to the safety of crops, vegetables and fruits, and the effective control of *Fusarium* is of great significance to ensure food safety [5].

At present, chemical fungicides are still the principal method to control *Fol*. A large number of chemical pesticides will cause soil and water pollution and seriously endanger human health. At the same time, the long-term use of chemical pesticides will cause drug resistance in *Fol*. Therefore, the use of environmentally friendly control methods is of particular importance [6,7]. Breeding tomato varieties that are resistant to *Fol* is a safe method, but it is time-consuming, and, if a new strain of *Fol* appears, breeding work also means failure. To date, three R genes against *Fol* have been identified (*I-1*, *I-2* and *I-3*). *I* gene specifically recognizes the effectors secreted by *Fol* and triggers an ETI (effector-triggered immunity) response [8]. In addition, biological control, as a safe, eco-friendly, efficient and low-cost method, has become the focus of *Fusarium* wilt control. At present, a variety of fungicides made up of antagonistic microorganisms, such as *Gliocladium virens*, *Aspergillus nidulans*, *Bacillus subtilis*, *Bacillus cereus*, *Streptomyces griseovirmis*, *Trichoderma harzianum* and *Pseudomonas chlororaphis*, have been commercialized [9,10]. The application of biological control agents significantly reduces the incidence rate of *Fusarium* wilt in tomato, watermelon, soybean, peanut, wheat, maize and rice [11,12,13].

These plant-growth-promoting rhizobacteria (PGPR) or antagonistic microorganisms are mainly isolated from the rhizosphere of plants [14]. The PGPRs or endophytic bacteria protect plants from pathogens by stimulating plants, inducing systemic resistance (ISR) or systemic acquired resistance to achieve the healthy growth of plants and a new dynamic balance of the rhizosphere environment [15,16,17,18]. It is worth noting that *P. polymyxa* is an important biocontrol PGPR that promotes plant growth through nitrogen fixation, solubilizes phosphate and produces plant phytohormones (IAA, GA3 and ABA) and relieves biotic stress by producing insecticidal or bactericidal substances, such as antimicrobial peptides, hydrolytic proteins, bacteriocins and lipopeptides [15,19,20,21]. Studies have shown that *P. polymyxa* can suppress the growth of pathogens, have a good inhibitory effect on tomato root-knot nematodes and can also prevent and control a variety of bacterial diseases, such as tobacco bacterial wilt, Chinese cabbage soft rot, cucumber angular leaf spot and cucumber *Fusarium* wilt, as well as a variety of fungal diseases, such as tobacco brown spot, tomato *Fusarium* wilt, rice blast and tomato gray mold [22,23,24,25]. However, the interaction between PGPRs and pathogens is also affected by many environmental factors, which affect the biocontrol potential of PGPR [3]. Furthermore, the interaction among host plants, pathogens and PGPRs is a multi-level complex interaction, which requires systematic and overall study to better understand the molecular biocontrol mechanism of PGPR against pathogens.

We previously isolated a biocontrol agent, *P.* *polymyxa* NSY50, from vinegar residue compost and used proteomics to reveal that NSY50 actively regulates the composition of the rhizosphere microbial community to induce a cucumber defense response against *Fusarium oxysporum* f. sp. *cucumerinum* [24,26,27]. At present, it has been identified that several PGPRs derived from *P.* *polymyxa* have certain inhibitory effects on tomato *Fusarium* wilt, such as the CF05 and HY96-2 strains [28,29]. In addition to *P.* *polymyxa*, there are many PGPRs that had better control effects on tomato *Fusarium* wilt. For instance, *B. amyloliquefaciens* SN16-1 has high control potential against *Fol* [30]. Compared with the infected control, *Azospirillum* *brasilense* and *Bacillus subtilis* can significantly reduce the severity of tomato *Fusarium* wilt disease [31]. The antagonistic activity of 24 strains isolated from eight rhizosphere soils of tomato planting zones in Bangladesh against *Fol* showed that *B.* *olei* and *B. methylotrophicus* caused almost 40% disease inhibition on Pusa Rubi and Ratan and could enhance the growth of tomato [32]. Six strains with the greatest antigenic activity against *Fol* strain 3 were identified from 126 strains of *Streptomyces*. Strains IC10 and Y28 could significantly mitigate the wilting symptoms of *Fusarium* wilt; increase the shoot length, fresh weight and dry weight and the peroxidase (PDX) activity and induce the expression of defense genes, such as *WRKY70*, *ERF1*, *LOX* and *TPX* [33]. The inhibition rates of *Pseudomonas fluorescens* PF15 and *Pseudomonas putida* PP27 on the growth and germination of *Fol* were 47% and 10%, respectively [34]. The *Bacillus aryabhattai* strain SRB02 can reduce the effects of *Fol* by regulating plant endogenous hormones (higher accumulation of SA and reduction in JA) and up-regulating the content of amino acids [3].

In this study, we evaluated the control effect of the agent *P.* *polymyxa* strain NSY50 on tomato *Fusarium* wilt caused by *Fol* and the growth-promoting effects on tomato. The transcriptome analysis of resistant responses to *Fol* in tomato mediated by *P.* *polymyxa* has not yet been studied. Therefore, a comparative transcriptome analysis was used to analyze the changes in differentially expressed genes (DEGs) in tomato root tissues induced by NSY50 after *Fol* infection and preliminarily analyzed the pathway of NSY50-mediated tomato resistance response to *Fusarium* wilt. Furthermore, qRT-PCR was used to verify the expression level of plant resistance genes in tomato. The function of the NAC transcription factor (TF) *SlNAP1* was verified using transgenic technology. In general, this study revealed the regulatory network of tomato resistance to *Fol* induced by *P. polymyxa* NSY50, confirmed that the *SlNAP1* gene could effectively increase the resistance of tomato plants to *Fol* and provided a basis for studying the antagonistic mechanisms against *Fusarium* wilt of tomato by *P.* *polymyxa* NSY50.

## 2. Results

### 2.1. P. Polymyxa NSY50 Can Effectively Relieve the Damage of Fol to Tomato

**Figure 1:**
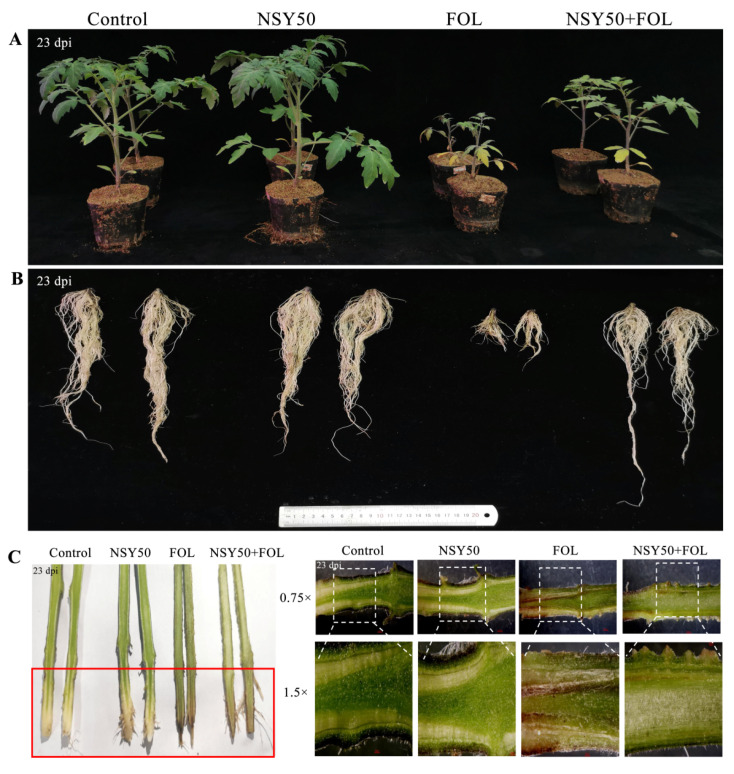
Effect of *P. polymyxa* NSY50 on phenotype of AC seedlings infected with *Fol*. (**A**) Plant phenotypic symptoms on the 23rd day after *Fol* inoculation; (**B**) root tissue of tomato plants on 23 dpi of different treatment; (**C**) longitudinal section of tomato plant stem.

We comprehensively evaluated the growth promotion and biocontrol efficiency of *P. polymyxa* NSY50 on susceptible tomato Ailsa Craig (AC). Phenotypic analysis showed that the plant height, root volume, fresh weight and dry weight of NSY50 treatment increased compared with the control, but the differences were not significant (Table 1). After inoculating *Fol*, NSY50 + FOL treatment could significantly enhance plant biomass compared with FOL treatment, in which plant height, fresh weight, dry weight and root volume improved by 76.40%, 90.46%, 148.74% and 306.99%, respectively. We could also clearly see that the tomato plants treated with FOL showed relatively weak growth and yellowing leaves, and the length and number of roots were significantly lower than thecontrol and NSY50 + FOL (Table 1, Figure 1A,B). The disease index survey showed that DI in the NSY50 + FOL treatment was significantly decreased by 28.34% compared with FOL (54.17%). In addition, we also found that the browning degree of vascular bundles in NSY50 + FOL was significantly lower than that in FOL, indicating that NSY50 could effectively delay the infection process of *Fol* (Figure 1C).

**Figure 2:**
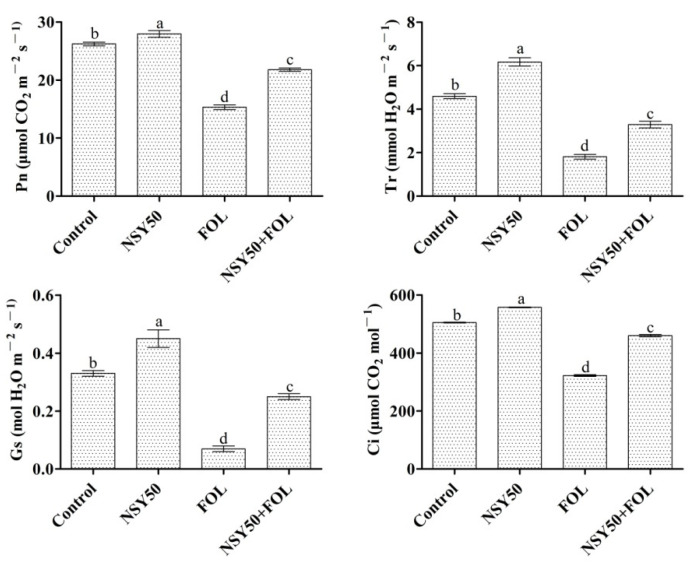
Photosynthetic parameters of tomato plants under different treatments. Different letters (a, b, c, and d) indicate statistically significant differences at *p* = 0.05 level.

Chlorophyll content and photosynthetic parameters were closely related to the changes in plant biomass. When NSY50 was applied alone, the chlorophyll content, Pn, Gs, Ci and Tr increased by 2.98%, 6.59%, 36.36%, 10.35% and 34.13%, respectively, compared with the control. Enhancement in photosynthesis promotes an increase in plant height and weight, indicating that NSY50 can promote the growth of tomato plants (Table 1). When *Fol* was inoculated alone (FOL treatment), the photosynthetic parameters and chlorophyll content of plants decreased significantly, the plant became shorter and the leaves turned yellow. However, when NSY50 was applied in advance and then inoculated with *Fol* (NSY50 + FOL treatment), chlorophyll content, Pn, Gs, Ci and Tr increased by 78.25%, 41.98%, 257.14%, 42.61% and 81.77%, respectively, compared with FOL (Figure 2). The plant growth status of plantstreated with NSY50 + FOL was significantly better than that of FOL, and only part of the leaflet of the first leaf was yellowed (Table 1, Figure 1A). After a comprehensive evaluation of the plant phenotype, photosynthesis and growth parameters, it was found that *P. polymyxa* NSY50 can promote the growth of tomato plants and can also effectively reduce the harmful impact of *Fol* on tomato.

### 2.2. Identification and Functional Annotation of DEGs in the Process of NSY50 Biological Control Fol

To elucidate the mechanism of NSY50 relieving the detrimental symptoms of *Fol* on tomato, a comparative transcriptome analysis was performed on the tomato root of AC treated with NSY50 or sterile water and in combination with *Fol* inoculation. After sequencing and quality control, a total of 57.20 Gb clean data were produced, the percentage of Q30 in each sample was higher than 92.97% and the GC content ranged from 43.51% to 44.13%. The sequence alignment rate between the sample and the reference genome was from 74.16% to 93.51%, and 71.16–90.36% of reads were uniquely mapped (Table S2). In addition, the correlation coefficients between different replicates were from 0.946 to 0.994, indicating that the samples had good repeatability (Figure S1). RNA-Seq data were of good quality and could be utilized to further analysis.

The gene expression levels in the samples of the control, FOL and NSY50 + FOL were determined. Compared with the control, a total of 7030 DEGs were identified in the NSY50 + FOL treatment, of which 3532 were up-regulated and 3,498 were down-regulated; there were 390 DEGs in NSY50 + FOL compared with FOL, of which 294 were up-regulated and 96 were down-regulated (Tables S3 and S4). By constructing a Venn diagram between the comparison groups, we found a total of 100 unique DEGs in the “NSY50 + FOL vs. FOL” group and the “NSY50 + FOL vs. Control” group, which may be closely related to NSY50 relieving the harmful effect of *Fol* on tomatoes (Figure S2). Additionally, we then annotated the functions of DEGs in several different databases, and the annotation results of each group of DEGs are shown in Table S5.

### 2.3. GO and KEGG Pathway Analysis of DEGs

To further explore the biochemical pathways of DEGs in NSY50 biological control *Fol*, GO and KEGG pathway analyses were performed. The results of the biological process of the GO analysis showed that the hydrogen peroxide catabolic process and response to oxidative stress were significantly enriched in the “NSY50 + FOL vs. Control” and “FOL vs. Control” groups, which may be related to the rapid outbreak of reactive oxygen species in tomato plants after *Fol* infection (Figure S3A,B). However, in the “NSY50 + FOL vs. FOL” group, we found that the significance of the above two processes and the number of enriched genes were clearly significantly reduced, while the serine/threonine phosphatase activity, glutamine metabolic process and abscisic-acid-activated signaling pathway were significantly enriched (Figure S3C). 

**Figure 3:**
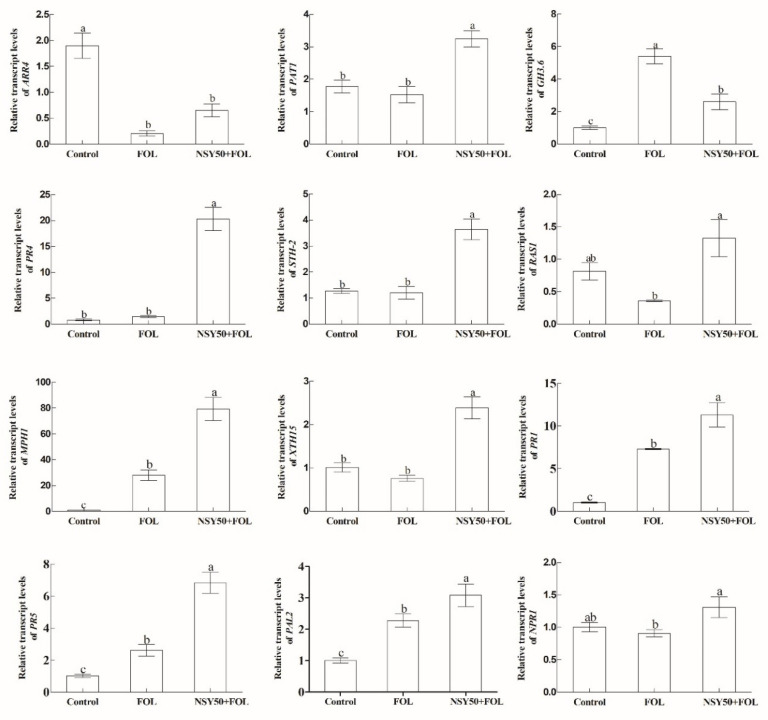
Effects of NSY50 on the relative transcript levels of 12 genes in tomato. Different letters (a, b, and c) indicate statistically significant differences at *p* = 0.05 level.

KEGG enrichment analysis showed that phenylpropanoid biosynthesis, starch and sucrose metabolism, glutathione metabolism, isoquinoline alkaloid biosynthesis, porphyrin and chlorophyll metabolism pathways were significantly enriched in the “NSY50 + FOL vs. Control” and “FOL vs. Control” groups. However, the enrichment levels of DEGs in these pathways were higher in the “NSY50 + FOL vs. Control” group than in the “FOL vs. Control” group (Figure S3D,E). Meanwhile, in the “NSY50 + FOL vs. Control” group, the plant–pathogen interaction pathway was also significantly enriched (Figure S3E). Meanwhile, we measured the indicator genes in the plant disease interaction and salicylic acid signal transduction pathways, such as *PR*, *NPR1*, *ARR4* and *PAT1*, using qRT-PCR. It was found that the expression levels of *PR*, *PAL2*, *MPH1*, *STH-2* and *PAT1* in the ‘NSY50 + FOL’ treatment were significantly higher than those in FOL and the control. In particular, *PR1* and *PR4* genes were highly expressed in the *PR* gene (Figure 3). In the “NSY50 + FOL vs. FOL” group, alkaloid biosynthesis, terpene biosynthesis, unsaturated fat acid biosynthesis and flavonoid biosynthesis were significantly enriched, and the synthesis and metabolites of these pathways were also closely related to plant disease resistance (Figure S3F). According to the differences among different comparison groups, when NSY50 was applied in advance, the plants could quickly mobilize their own defense responses to resist the infection of the pathogen.

### 2.4. Identification of NAC Transcription Factors in Tomato after Fol Infection

**Figure 4:**
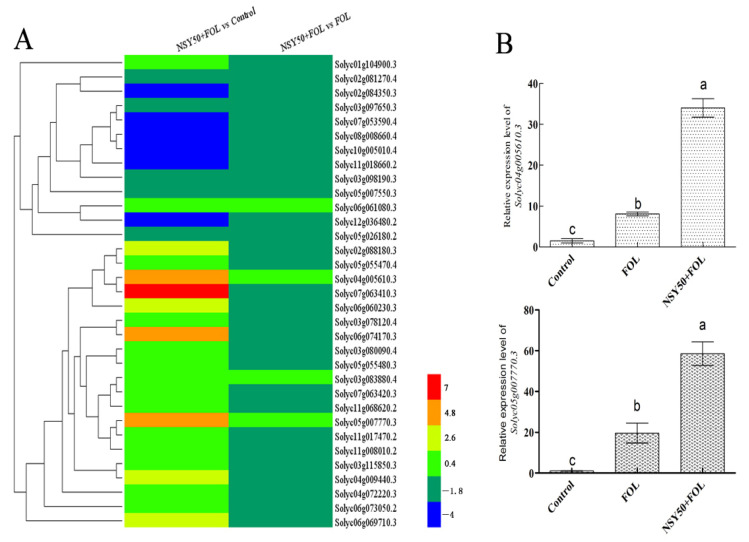
*SlNAP1* and *SlNAP2* were strongly induced by *Fol.* (**A**) Heatmap of 33 differentially expressed NAC transcription factors; (**B**) relative expression levels of *SlNAP1* and *SlNAP2* under different treatments. Different letters (a, b, and c) indicate statistically significant differences at *p* = 0.05 level.

Our previous study found that NAC transcription factor *SlNAP1* can increase salt tolerance by modulating ion homeostasis. In order to analyze the response of tomato NAC genes to *Fol* infection, NAC transcription factors in the transcriptome data of the control, FOL and NSY50 + FOL were statistically analyzed. We identified 33 differentially expressed NAC TFs and their expression ratios after *Fol* infection (Figure 4A, Table S6). Compared with the ‘Control’, the expression of four NAC genes (*Solyc04g005610.3*, *Solyc05g007770.3*, *Solyc06g074170.3* and *Solyc07g063410.3*) was significantly up-regulated in the ‘NSY50 + FOL’ treatment (4.87 < Log_2_FC < 7.31). Moreover, when compared with the ‘FOL’ treatment, only *Solyc04g005610.3* (*SlNAP2*) and *Solyc05g007770.3* (*SlNAP1*) were up-regulated (0.77 < Log_2_FC < 1.25). We measured the expression of these two genes in the three treatments. As shown in Figure 4B, the expression of the *Solyc05G007770.3* gene significantly increased and reached the maximum in the ‘NSY50 + FOL’ after *Fol* inoculation, which was 58.2 times higher than that in the control. The expression of *Solyc04g005610.3* was also significantly increased, but the expression level was slightly lower than that of *Solyc05g007770.3*.

### 2.5. SlNAP1 Promotes Plant Growth and Relieves the Harmful Effect of Fol on Tomato

**Figure 5:**
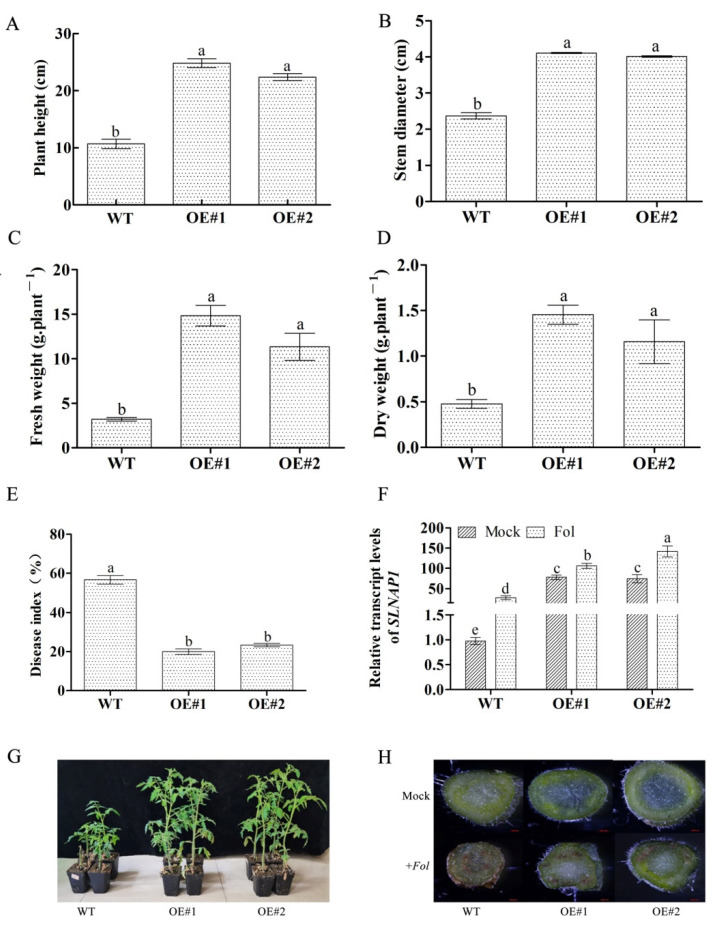
Effects of *SlNAP1* overexpression on *Fusarium* wilt resistance after *Fol* inoculation. (**A**–**D**) Phenotypic symptoms (plant height, stem diameter, fresh weight and dry weight) of plants at 21dpi after *Fol* inoculation; (**E**) photosynthetic parameters of overexpressed plants; (**F**) transcript abundance of *SlNAP1* gene in transgenic plants; (**G**,**H**) plant phenotype and stem cross section at 21 dpi. Different letters (a, b, c, d, and e) indicate statistically significant differences at *p* = 0.05 level.

To study the effect of the *SlNAP1* gene on the retardation or attenuation of the harmful impact of *Fol* on tomato, we performed a *SlNAP1* overexpression experiment and obtained two homozygous lines: OE#1 and OE#2. We inoculated the four-leaf-old overexpression plants with *Fol* and found that there was no significant difference in plant height, stem diameter, fresh weight and dry weight between the two *SlNAP1* overexpressed tomatoes at 21 dpi, but they were significantly higher than those of WT plants, with average values of 2.21, 1.71, 3.69 and 2.66 higher than the control (Figure 5A–D). The measurement of photosynthetic parameters showed that the GS and Ci of overexpression plants were not significantly different from WT, but Pn and Tr were significantly higher than WT (Figure S4). Then, we analyzed the correlation between the photosynthetic parameters, fresh weight and dry weight of WT, OE#1 and OE#2. The results showed that there was a significant correlation between fresh weight and dry weight (R^2^ = 0.969). Both fresh weight and dry weight were negatively correlated with Ci, and the correlation coefficients were −0.781 and −0.792, respectively. Fresh weight and Pn were significantly positively correlated with Tr, and the correlation coefficients were 0.686 and 0.853, respectively (Table S7). The transcript abundance of the *SlNAP1* gene showed that the expression of *SlNAP1* in two overexpressed plants was significantly higher than that in WT, and the expression of *SlNAP1* in overexpressed plants was significantly increased by 3.8 and 5.1 times compared to WT after *Fol* inoculation (Figure 4F). Combined with the observation of plant phenotypes of OE#1 and OE#2, we observed that, after *Fol* inoculation, the growth of OE#1 and OE#2 plants was significantly better than that of WT, and the browning degree of vascular bundles in overexpressed plants was significantly lower than that of WT (Figure 4G,H). The results showed that the harmful effect of *Fol* on tomato was effectively relieved. We analyzed the disease index (DI) of two *SlNAP1* overexpression plants inoculated with *Fol* and found that the DI of OE#1 and OE#2 significantly decreased by 38.34% and 33.34%, respectively, compared with the control (Figure 4E).

## 3. Discussion

Tomato growth requires a good ecological environment, and the plant rhizosphere, water, oxygen, mineral elements and beneficial microorganisms must maintain a healthy dynamic balance. However, such an ideal state is difficult to achieve. The reality is that the above-ground tissues and roots of tomato are always disturbed by pathogenic microorganisms. In production, we usually use chemical insecticides or fungicides to control these harmful pathogens. However, excessive application of chemical pesticides will pollute the environment and bring indirect harm to people or animals. 

PGPR, such as *P. polymyxa*, *B. amyloliquefaciens*, *B. Olei*, *B. aryabhattai*, *Pseudomonas putida* and *B. subtilis*, as important beneficial bacteria that can not only promote plant growth but also act asbiological control agents to control soil-borne diseases, have been widely discovered and applied [3,19,30,31,33,34]. *P. polymyxa* strains are well known for their plant-growth-promoting (PGP) properties and as biocontrol agents against harmful plant pathogens [25]. *P. polymyxa* strains were isolated from crop rhizosphere and internal tissues, such as strains B1, B2, CF43, PMD216 and PMD112 isolated from wheat rhizosphere [35,36,37]; BMP-11, BRF-1 and SQR-21 were isolated from cucumber, soybean, watermelon and muskmelon, respectively [38,39] and JSa-9, SR04-02, SR04-16, EG2 and EG14 were isolated from soil [40,41]. These strains showed antagonistic activity against pathogenic fungus (such as *Fusarium oxysporum* f. sp. *niveum*, *Pseudomonas syringae* pv., *Lachrymans* and *Acidovorax* *avenae* subsp. *citrulli*) and may be promising biocontrol agents. *P.* *polymyxa* NSY50 used in this experiment has an obvious inhibitory effect on the *Fusarium* wilt of cucumber. Therefore, we aimed to explore whether NSY50 could also reduce the harmful effect of *Fol* on tomatoes. We conducted a confrontation assay between NSY50 and *Fol* in the PDA medium. It was obvious that there was no *Fol* hypha at the inoculation site of NSY50 (Figure S5). The plant height, dry weight, fresh weight and root volume of AC plants treated with NSY50 were significantly better than the control and FOL treatment, indicating that NSY50 can promote an increase in tomato plant biomass. At the same time, NSY50 could significantly reduce the incidence rate of *Fusarium* wilt after inoculation with *Fol*. The preliminary results showed that NSY50 was a new PGPR in *P. polymyxa* and could be used as a biocontrol agent to control tomato *Fusarium* wilt (Table 1, Figure 1).

At present, it is known that *P. polymyxa* strains CF05, SC09-21, SR04-02 and SR04-16 can improve plant growth, suppress tomato *Fusarium* wilt and *Fusarium* crown, induce the systemic resistance of tomato plants, stimulate the release of plant defense enzymes and protect tomato from pathogens [25,29,41]. In light of this, how do *P. polymyxa* strains NSY50 promote plant growth and suppress tomato *Fusarium* wilt?

Based on the determination of the chlorophyll content and photosynthetic parameters, when NSY50 was pretreated, the photosynthetic capacity of the plant was superior to the control and significantly higher than that of the FOL treatment (Table 1 and Figure 2), indicating that NSY50 can improve the photosynthetic efficiency of tomato seedlings under *Fusarium* wilt stress, promote the photosynthetic carbon assimilation capacity and reduce the inhibitory effect of *Fusarium* wilt stress on the growth of tomato seedlings. These results were consistent with the research conclusions of Du and Ahammed [42,43], who found that PGPR could effectively improve the photosynthetic performance of cucumber. Then, we performed transcriptome analysis of ‘NSY50 + FOL’, ‘FOL’ and control treatments and found that NSY50 could improve the efficiency of sucrose metabolism in tomato. Compared with the control, the expression was significantly up-regulated of sucrose phosphate synthase gene (*Solyc09g092130.3*) and sucrose synthase gene (*Solyc01g102840.3*, *Solyc07g04250.3* and *Solyc12g009300.3*) in the starch and sucrose metabolism pathway (ko00500) and phosphomannomutase gene (*Solyc05g046340.2*) in the fructose and mannose metabolism pathway (ko00051), thereby increasing the content of sucrose and starch in the plant and providing a material and energy basis for the normal metabolism of plants. Previous studies have shown that high levels of soluble sugar, fructose, sucrose and starch in plants not only provide sufficient energy for plant growth but also play a positive role in balancing intracellular osmotic potential and improving plant resistance [44,45]. This was also consistent with our result in this study that, when NSY50 was pretreated, the incidence rate of *Fusarium* wilt of tomato seedlings significantly decreased by 28.34% compared to without NSY50 (Table 1). In addition, among the disease-resistance-related pathways enriched by DEGs, we found that *PR1* genes*Solyc09g007010.1* and *Solyc09g007020.1* and *ChiB* genes *Solyc01g097280.2*, *Solyc07g009500.3* and *Solyc07g009510.1* in the MAPK signaling pathway (ko04016) were significantly up-regulated, and they played a positive role in enhancing the plant pathogen defense. We also found that TG TFs (*Solyc10g08521.2*) and the *PR1* gene in the SA metabolic pathway of the plant hormone signal transduction pathway (ko04075) were also significantly up-regulated. Additionally, in the SA biosynthesis isochorismate synthase pathway (ko01851), isochorismate synthase (ICS) genes *Solyc10g083270* and *Solyc02g085180.5.1* enhanced disease susceptibility 1 (EDS1) gene *Solyc06g071280.4*, and *PAL* genes *Solyc10g011925.1*, *Solyc03g042560.3*, *Solyc12g096190.2* and *Solyc05g056170.3* in the phenylalanine ammonia-lyase pathway (ko10775) were significantly up-regulated. A large amount of SA synthesized by plants plays a positive role in resisting *Fol* infection. However, approximately 10% of the SA biosynthesis induced by pathogens in *Arabidopsis thaliana* was due to the phenylalanine ammonia-lyase pathway, while 90% of SA was synthesized by the isochorismate synthase pathway [46].

Transcriptional regulation of gene expression plays an important role in plant adaptation to the environment and resistance to stress. TFs are a class of important regulatory genes that regulate gene expression. They activate or repress the transcriptional expression of target genes by binding to specific DNA sequences in the promoters of target genes. Combined with previous studies on NSY50, we found that NAC TFs, especially *SlNAP1* and *SlNAP2* genes, were significantly up-regulated by 2^5.95^ and 2^6.08^ times in *Fol* infection after pretreatment with NSY50 (Table S6). Recent studies have shown that NAC TF family genes play an important role in the disease resistance response of plants to pathogens. For example, the expression of *OsNAC19* in rice is induced by infection of *Magnaporthe grisea* and disease resistance signaling molecules MeJA, ABA and ETH [47]; Jensen found that overexpression of the *ATAF1* gene enhanced resistance to barley powdery mildew, while *ataf1* mutant plants decreased resistance to powdery mildew [48]. *Arabidopsis ANAC019* and *ANAC055* genes participate in JA-mediated defense response and regulate the expression of JA-induced defense genes *VSP1* and *LOX2*. The overexpression of the *ANAC019* and *ANAC055* genes in *Arabidopsis thaliana* can enhance resistance to *Botrytis cinerea* [49]. In this study, in order to elucidate the function of NAC TFs in the response to tomato *Fusarium* wilt, we constructed two overexpression plants of NAC TFs: *SlNAP1* and *SlNAP2*. According to the plant phenotype of the two *SlNAP1* overexpression homozygous lines OE#1 and OE#2 inoculated with *Fol*, we found that OE#1 and OE#2 plants grew better than the control, and the incidence rate of *Fusarium* wilt was also significantly lower than that of the control (Figure 5). At present, we have not obtained *SlNAP2* overexpression plants, but, fortunately, we have just obtained a few heterozygous T0 generation tomato plants with the single and double knockout of *SlNAP1* and *SlNAP2* genes. After obtaining the homozygous lines, we will carry out subsequent salt stress and phenotype identification. However, previous studies have confirmed that the *SlNAP1* overexpression plants showed better resistance to *Pseudomonas syringae* PV. DC3000 and *Ralstonia solanacearum* [50]. Therefore, combined with the findings of this study, the *SlNAP1* gene should also play a positive role in alleviating the detriment of tomato to *Fusarium* wilt.

## 4. Materials and Methods

### 4.1. Plant Material, Microbial Culture and Inoculation Treatment

Tomato seeds (Ailsa Craig, AC) were sterilized with 2.75% NaClO for 3 min, washed 3 times with sterile water and then sown in seedling nursing containers filled with sterilized vermiculite (3.15′′D × 3.15′′W × 3.54′′ inches, square plastic pots). Three-leaf-old tomato plants with the same growth potential were selected for inoculation. During the process of tomato cultivation, Hoagland nutrient solution was applied. The photoperiod was 16 h (light): 8 h (dark), the temperature was 25 °C, the relative humidity was 75% and the photosynthetic photon flux density (PPFD) was 200 µmol m^−2^·s^−1^. *P.* *polymyxa* NSY50 was grown on TSB medium at 28 °C for three days while shaking at 160 rpm and then centrifuged at 6000 rpm for 5 min. The supernatant was discarded and suspended in sterile water, and the concentration was adjusted to 1 × 10^8^ CFU/mL. *Fol* was cultured in PDB for 7 days, filtered by a gauze and adjusted to 1 × 10^7^ spores per mL with sterile water. Tomato seedlings were inoculated as follows: (1) control, 50 mL sterile water; (2) FOL, 50 mL spore suspension of *Fol*; (3) NSY50, 50 mL cell suspension of NSY50; (4) NSY50 + FOL, 50 mL NSY50 cell suspension was inoculated, and, 3 days later, 50 mL *Fol* was inoculated. *Fol* or NSY50 suspension was poured into the substrate of the root of the tomato plants.

### 4.2. Biocontrol Evaluation of NSY50

At 23 days post-inoculation (dpi), we measured the disease index (DI) values, tomato growth parameters and leaf photosynthetic indexes of different treatments. DI was calculated referring to the methods of Akram [51] and Cachinero [52]. The scoring criteria of wilting and DI are presented in Supplementary File S1. The data of tomato plant height, fresh weight and dry weight were recorded. Root volume was determined using the WinRHIZO STD4800 LA2400 root analysis system (Regent Instruments Inc., Québec, QC, Canada). The chlorophyll content of tomato leaves was measured using a SPAD-502 chlorophyll-meter (Minolta Camera Co. Ltd., Osaka, Japan). Photosynthetic characteristics, such as Pn, Gs, Ci and Tr, were determined using LI-6400XT (LI-COR, Inc., Lincoln, NE, USA) at 10 to 11 a.m. as described by Zhang [53].

### 4.3. RNA Extraction and qRT-PCR Analysis

After 24h of inoculation with water (control) or *Fol*, the root tissues of tomato plants in each treatment were soaked in liquid nitrogen. Total RNA was extracted from root tissues using the TRIzol method (Thermo Fisher Scientific, Waltham, MA, USA) and reversed into cDNA using the Superscript IV reverse Transcriptase kit (Thermo Fisher Scientific, Waltham, MA, USA). Fourteen genes related to plant disease resistance and plant hormone signal transduction, such as *PR1*, *NPR1*, *PAL2*, *PR4* and *RAS1*, underwent procedure for qRT-PCR that applied a CFX96^TM^ Real-Time System (Bio-Rad, Hercules, CA, USA) using the PowerUp™ SYBR™ Green Master Mix (Thermo Fisher Scientific, Waltham, MA, USA). The qRT-PCR primers are shown in Table S1, and the relative expression was calculated using the 2^−ΔΔCt^ method [54].

### 4.4. Sequencing Library Construction, Sequencing, Quality Control and Mapping

High-quality RNA from the root tissues of the control, FOL and NSY50 + FOL treatments was used to construct sequencing libraries. Based on the sequencing using the synthesis (SBS) technology, the cDNA library was sequenced on the Illumina Hiseq4000 platform by Biomarker Technologies Corporation (Beijing, China) and the read length was PE150. Ewing’s method was used to evaluate the basic quality of raw reads [55]. Clean reads were obtained by filtering out low-quality reads, as well as evaluating the GC content and Q30 value. Clean reads were mapped to the tomato reference genome Heinz1706 using HISAT2 software 4.0 (https://solgenomics.net/ftp/genomes/Solanum_lycopersicum/Heinz1706/assembly/build_4.00/, accessed on 18 November 2021) [56].

### 4.5. Screening of DEGs, Annotation, GO and KEGG Analysis

The FPKM value was used to measure the expression level of transcripts, and the DESeq2 software package was used to calculate the differential expression of transcripts between the samples; the transcripts with fold change ≥2 or ≤−2 and *p*-adjusted value <0.01 were considered as the DEGs [57]. The DEGs were annotated in COG, KOG, NR and Pfam databases. GO and KEGG enrichment was performed using Go-seq and KOBAS 3.0 software, respectively, and a *p*-value < 0.01 was considered to be significantly enriched [58,59]. NAC TFs are involved in the regulation of plant growth and development and play an important role in the plant disease resistance response to pathogens. Therefore, the expression levels of NAC TFs significantly enriched in DEGs were determined, and the identification of overexpression plants via inoculation was carried out.

### 4.6. Construction and Resistance Evaluation of Overexpression and Knockout Plants

The CDS sequences of the *SlNAP1* (*Solyc05g007770.3*) and *SlNAP2* (*Solyc04g005610.3*) genes were amplified with specific primers (Table S1), and the PCR product was digested by AscI and KpnI and cloned into a PFGC1008-HA vector. The CRISPR/Cas9 vector was kindly provided by Shi Lu (Institute of Agricultural Product Safety and Nutrition, Jiangsu Academy of Agricultural Sciences, China). The targetsof PAM-guide sequences for knockout *SlNAP1* (*Solyc05g007770.3*), *SlNAP2* (*Solyc04g005610.3*) and *SlNAP1*+*SlNAP2* genes were designed using CRISPR-P 2.0 [60]. Transgenic tomato plants were obtained according to Abraham’s protocol [61]. We selected two homozygous lines, OE-*SlNAP1*-1 and OE-*SlNAP1*-2, with a high expression of the *SlNAP1* gene for subsequent experiments. Phenotypic analysis was performed on AC and transgenic plants after the inoculation of *Fol*.

### 4.7. Statistical Analysis

Each treatment had 30 tomato seedlings, and three biological replicates were performed. Analysis of variance was performed using SPSS (SPSS Inc., Chicago, IL, USA), and significance analysis was conducted using an LSD test with a confidence level of 95%; the different letters indicate significant differences between treatments. All histograms were performed on Graphpad Prism 5 software (GraphPad, San Diego, CA, USA).

## 5. Conclusions

In this study, we identified a highly efficient PGPR strain, *P. polymyxa* NSY50, which can improve the growth and photosynthetic performance of tomato seedlings and promote the ability of tomato sucrose metabolism and fructose and mannose metabolism under *Fol* stress. Transcriptome analysis showed that NSY50 could also improve the alkaloid biosynthesis, terpene biosynthesis and unsaturated fat acid biosynthesis abilities of tomato plants and play a positive role in reducing the damage caused by *Fol* infection and enhancing the disease tolerance of seedlings. Through transgenic validation, we identified an important tomato NAC transcription factor, *SlNAP1*, which may play an active role in relieving the detrimental symptoms induced by *Fol*.

## Figures and Tables

**Table 1:** Effects of *P. polymyxa* stain NSY50 on the growth, chlorophyll content and DI of tomato AC seedlings under *Fol* stress.

Treatment	Growth Parameters				Chlorophyll Content/SPAD	DI/%
	Plant Height/cm	Fresh Weight/g	Dry Weight/g	Root Volume/cm^3^		
Control	19.72 ± 0.21 a	24.42 ± 1.03 a	2.32 ± 0.12 a	13.77 ± 1.05 a	50.30 ± 0.52 a	0.00
NSY50	20.38 ± 0.26 a	25.36 ± 1.18 a	2.43 ± 0.15 a	13.97 ± 1.39 a	51.80 ± 0.31 a	0.00
FOL	6.47 ± 0.46 c	4.75 ± 0.19 c	0.40 ± 0.04 c	1.30 ± 0.20 c	22.07 ± 0.88 c	54.17 ± 1.80 a
NSY50 + FOL	11.41 ± 0.33 b	9.05 ± 0.65 b	0.99 ± 0.06 b	5.29 ± 0.32 b	39.33 ± 0.64 b	25.83 ± 2.20 b

Note: values are means of replicates ± SE; different letters indicate statistically significant differences at *p* = 0.05 level.

## Data Availability

The raw data of transcriptome sequencing were stored in the SRA database of NCBI (http://www.ncbi.nlm.nih.gov/sra, accessed on 30 August 2022). The accession numbersare: SRR21098351, SRR21098350, SRR21098349, SRR21098348, SRR21098347, SRR21098346, SRR21098345, SRR21098344 and SRR21098343.

## Supplementary Materials

The following supporting information can be downloaded at: https://www.mdpi.com/article/10.3390/ijms231810907/s1.
