# Supplementary material for: Comparative Transcriptome Analysis and Genetic Methods Revealed the Biocontrol Mechanism of Paenibacillus polymyxa NSY50 against Tomato Fusarium Wilt
Source: Int J Mol Sci. 2022 Sep 18;23(18):10907. doi: 10.3390/ijms231810907 (PMC9501285; doi:10.3390/ijms231810907)
Supplement: Supplementary file 1 [file ijms-23-10907-s001.zip › Supplementary File S1. Scoring criteria of wilting and DI.pdf]

Attachment 1. Scoring criteria of wilting and disease index (DI)

Scoring of tomato wilting symptoms in tomato caused by *FOL* infection (score 0-3) was conducted by using following criteria developed by Epp (1987) and the scoring of wilt symptoms is as follows:

Wilt score	Symptoms
0	No wilt symptom
1	Less than 25% plant parts turned yellow
2	Yellowing and browning covered less than 50% plant parts
3	Infected plant parts turned brown and died, hence covered more than 50% plant parts

Disease Index (DI) was calculated using the following equation:

DI=[(Ni×Si)/(N×S)]×100%

(Note: Ni, number of tomato plants with wilt symptoms; Si, value of the score of symptoms; N, total number of tested tomato plants; S, the highest value of score of symptoms.)

Overall responses of the tested tomatoes against *Fusarium* wilt was established using the following criteria:

DI value	Disease evaluation
0	Rmmune
1-20%	Resistant
21-40%	Moderately susceptible
41-70%	Susceptible
71- 100%	Very susceptible
