# Supplementary figures and images for: Comparative Transcriptome Analysis and Genetic Methods Revealed the Biocontrol Mechanism of Paenibacillus polymyxa NSY50 against Tomato Fusarium Wilt
Source: Int J Mol Sci. 2022 Sep 18;23(18):10907. doi: 10.3390/ijms231810907 (PMC9501285; doi:10.3390/ijms231810907)

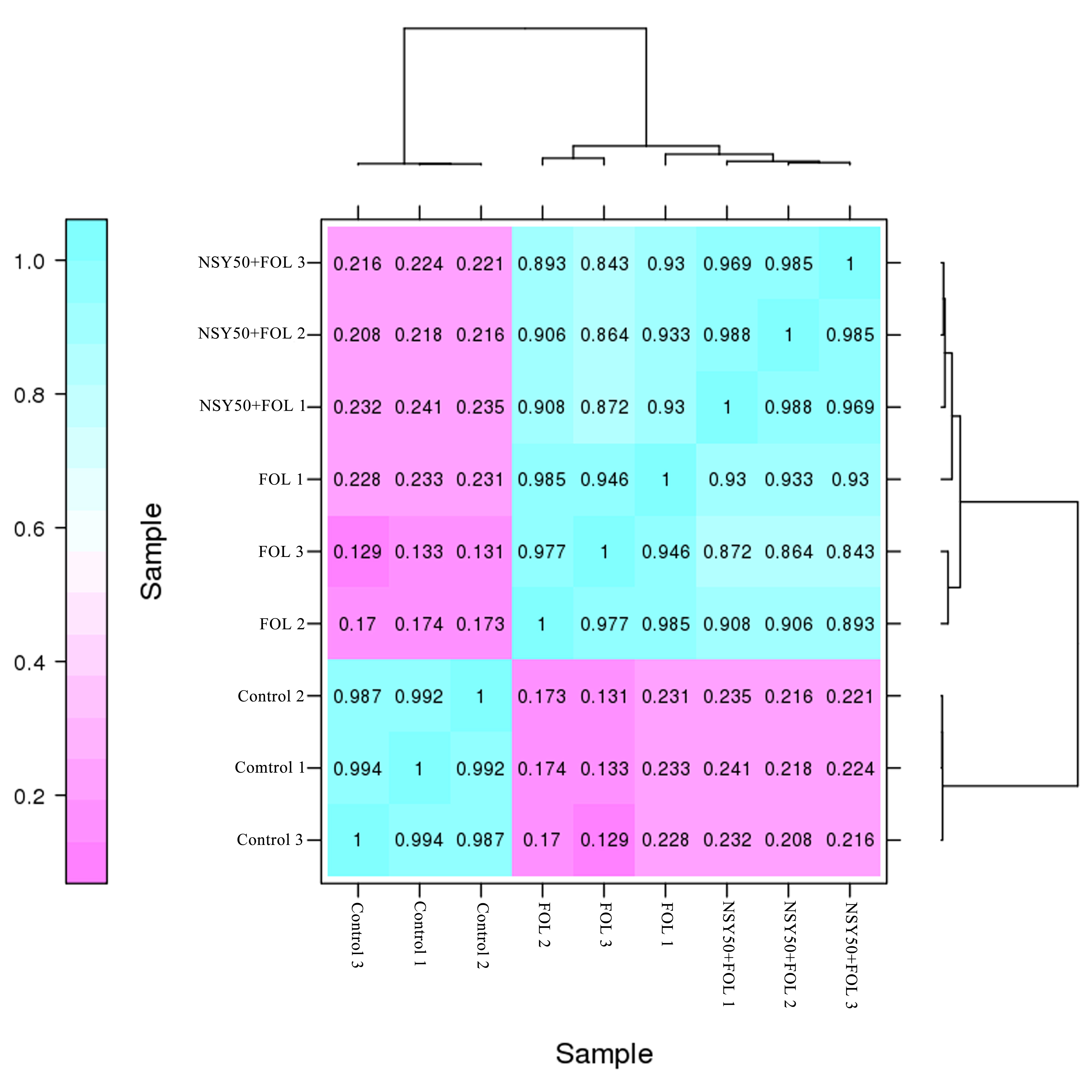

Supplement: Supplementary file 1 [file ijms-23-10907-s001.zip › Figure S1. Correlation analysis of RNA-Seq data between samples.jpg]

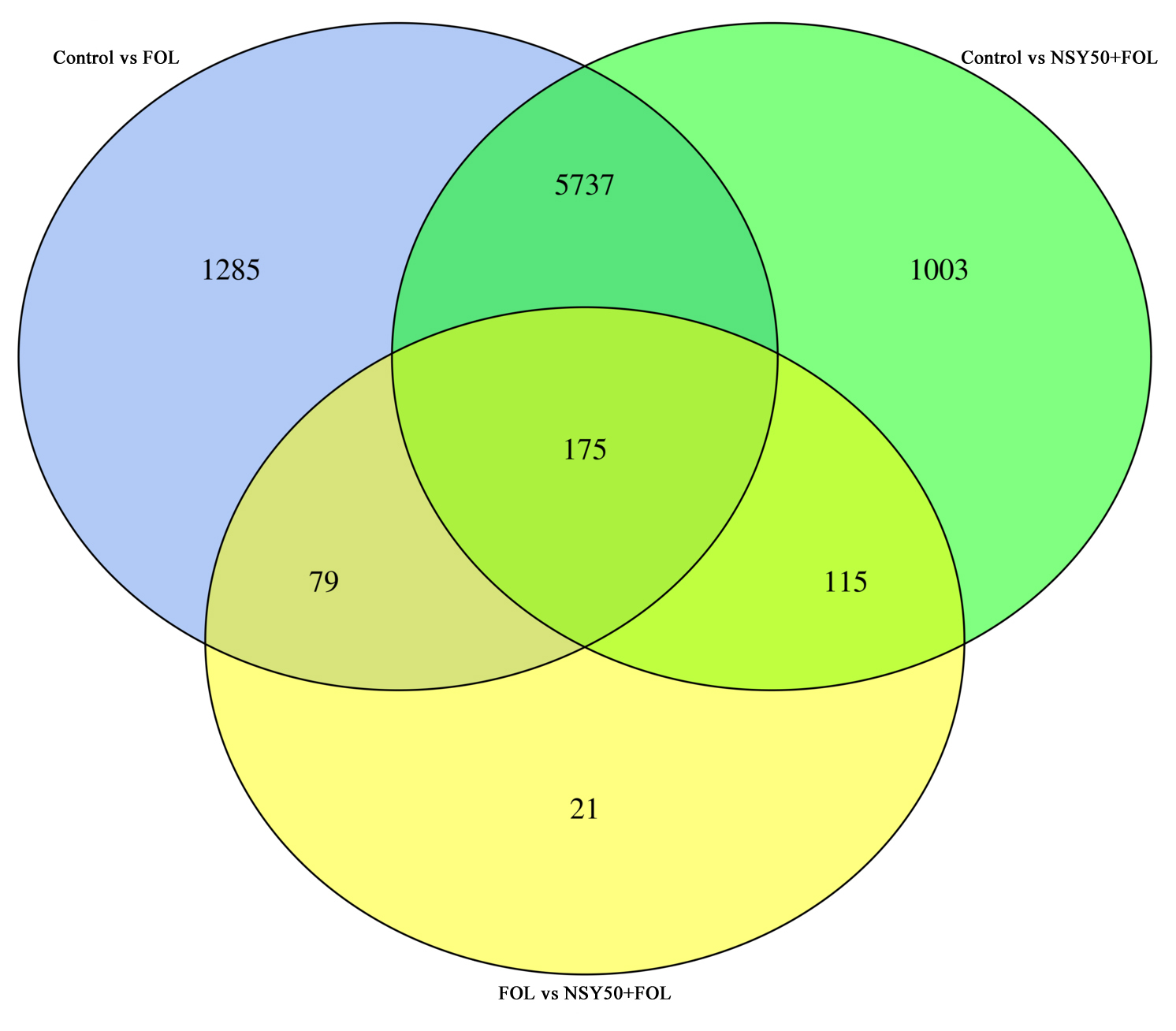

Supplement: Supplementary file 1 [file ijms-23-10907-s001.zip › Figure S2. Venn diagram of DEGs between different comparison groups.jpg]

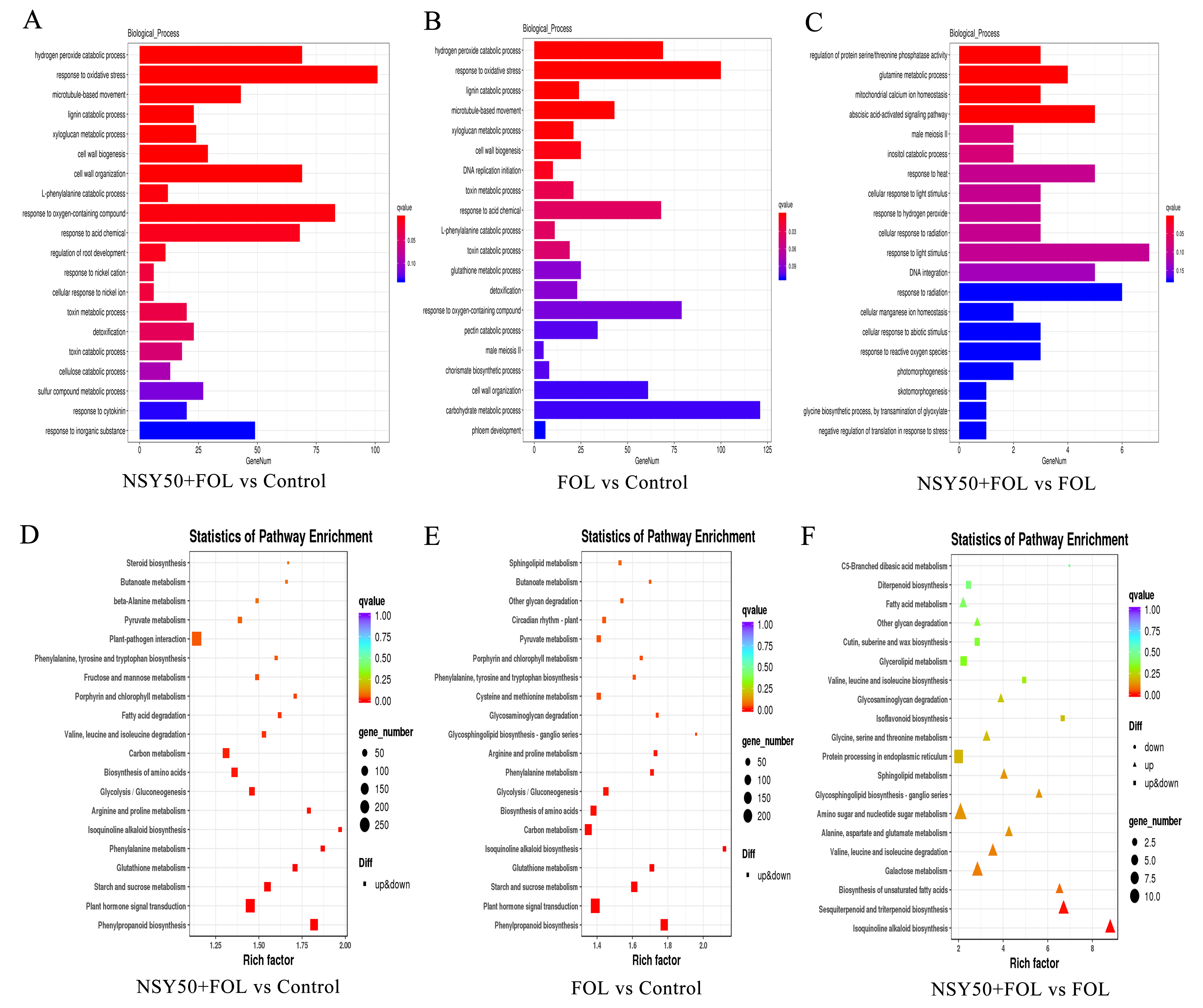

Supplement: Supplementary file 1 [file ijms-23-10907-s001.zip › Figure S3. GO and KEGG pathway enrichment analysis of DEGs.jpg]

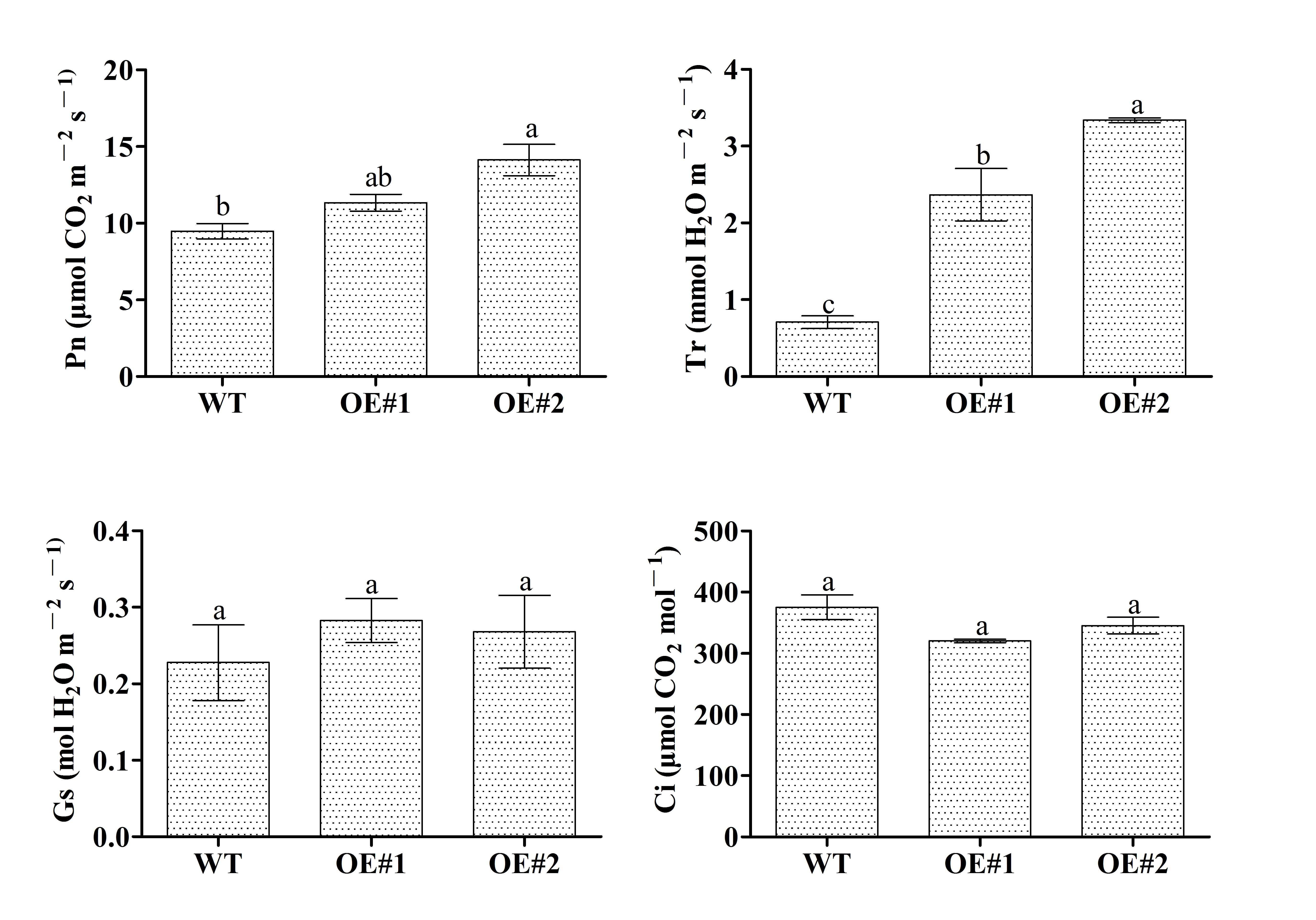

Supplement: Supplementary file 1 [file ijms-23-10907-s001.zip › Figure S4. Photosynthetic parameters of SlNAP1 gene overexpression plants.jpg]

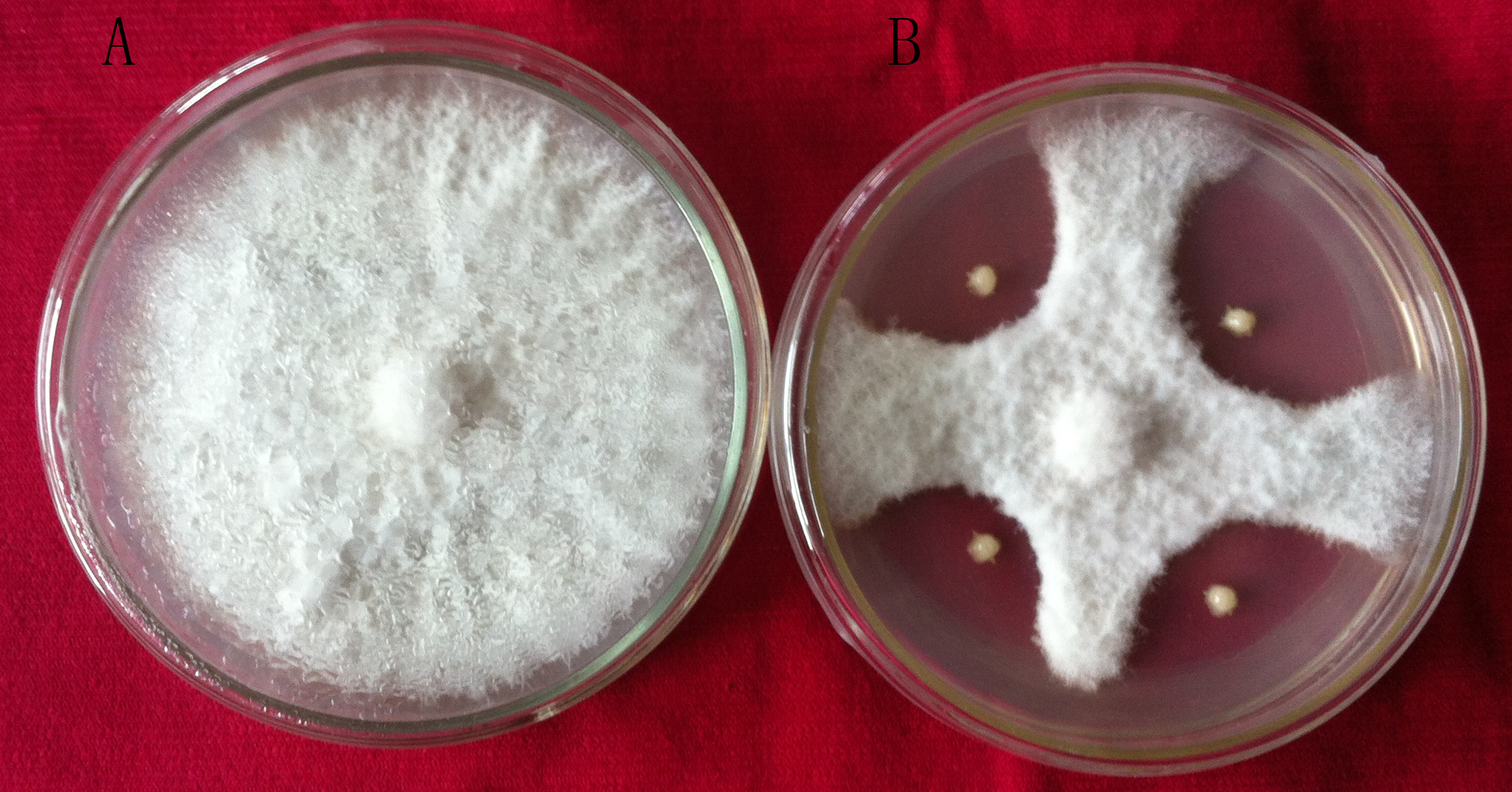

Supplement: Supplementary file 1 [file ijms-23-10907-s001.zip › Figure S5. Antagonistic effect of NSY50 against Fol. a, control; b, Co-inoculated.jpg]
